# Antidiabetic Activity of Gold Nanoparticles Synthesized Using Wedelolactone in RIN-5F Cell Line

**DOI:** 10.3390/antiox9010008

**Published:** 2019-12-21

**Authors:** Vinayagam Ramachandran, Mariadoss Arokia Vijaya Anand, Ernest David, Karthikkumar Venkatachalam, Shalini Vijayakumar, Vijayalakshmi Sankaran, Agilan Balupillai, Casimeer C. Sangeetha, K. M. Gothandam, Venkata Subbaiah Kotakadi, Alaa Ghidan, Tawfiq Al Antary, Baojun Xu

**Affiliations:** 1 Department of Biotechnology, Thiruvalluvar University, Vellore 632115, India; mavijaibt@gmail.com (M.A.V.A.); shalini9296@gmail.com (S.V.); vijayalakshmisk13@gmail.com (V.S.); agilanphd@gmail.com (A.B.); 2 Department of Pharmacology and Therapeutics, College of Medicine of Health Sciences, United Arab Emirates University, Al Ain 17666, UAE; karthikjega@gmail.com; 3 Department of Physics, Sri Padmavati Mahila Visvavidyalayam (Women University), Andhra Pradesh 517502, India; carosangee@gmail.com; 4 Vellore Institute of Technology (VIT), School of Biological Sciences and Technology, Vellore 632104, India; gothandam@gmail.com; 5 DST PURSE Centre, Sri Venkateswara University, Tirupati 517502, Andhra Pradesh, India; kotakadi72@gmail.com; 6 Pharmacy School, Aqaba University of Technology, Aqaba 77110, Jordan; alaa_ghidan@yahoo.com; 7 Department of Plant protection, Faculty of Agriculture, University of Jordan, Amman 11942, Jordan; awfiqalantary@yahoo.com; 8 Food Science and Technology Program, Beijing Normal University–Hong Kong Baptist University United International College, Zhuhai 519087, Guangdong, China; baojunxu@uic.edu.hk

**Keywords:** antidiabetic, wedelolactone, gold nanoparticles, di-(2-ethylhexyl) phthalate, RIN-5F cell line, cytotoxicity

## Abstract

We synthesized the gold nanoparticles (AuNPs) using wedelolactone (WDL) and characterized them using UV-visible spectroscopy, fourier transform infrared spectroscopy (FTIR), X-ray diffraction (XRD), scanning electron microscopic (SEM), transmission electron microscopic (TEM), energy dispersive X-ray diffraction, and atomic force microscopic (AFM) studies. The electronic spectrum exhibited an absorption peak at 535 nm. The FT-IR results proved that WDL was stabilized on the surface of AuNPs by acting as a capping or reducing agent. The crystalline structure was affirmed by XRD pattern and the spherical shape of WDL-AuNPs was evidenced by SEM, TEM, and AFM. The synthesized WDL-AuNPS were evaluated for anti-diabetic activity in pancreatic RIN-5F cell lines. *In vitro* results showed that WDL-AuNPs did not only improve the insulin secretion affected by di-(2-ethylhexyl) phthalate (DEHP), but also the cell viability in RIN5F cells. WDL-AuNPs treatment modulates the pro-apoptotic proteins and anti-apoptotic proteins expression to prevent the cells undergoing apoptosis in DEHP-exposed RIN-5F cells. The exposure of DEHP causes an increase in ROS production and lipid peroxidation levels. The free radical scavenging and antioxidant properties of WDL-AuNPs increase the deleterious effect caused by DEHP. On the other side, WDL-AuNPs increase mRNA expressions of insulin-signaling proteins in RIN-5F cells. This study concludes that WDL-AuNPs can be successfully used to regulate the expression of Bcl-2 family proteins, reduce lipid peroxidation, and to improve the secretion of antioxidants and insulin through the GLUT2 pathway in RIN-5F cell lines.

## 1. Introduction

Nanotechnology deals with materials having the size of 1 to 100 nm at least in any one of the dimensions. Nowadays, a variety of green nanoparticles (NPs) with defined physico-chemical, biological, medical, and environmental sciences. Researchers show utmost interest in nano-materials because of the interesting morphological and physicochemical characteristics and promising biomedical applications of these kind of materials [1,2]. The gold nanoparticles (AuNPs) have received great attention in imaging, drug delivery, thermotherapy, and also in bio-sensing [3]. As far as the biosynthesis of AuNPs is considered, the factors, including capping and reducing agents and solvent, are very influencing.

The utilization of raw plant extracts, phytochemicals, and certain microorganisms is a greener and alternative approach to chemical synthesis of NPs [4]. AuNPs of different colors (orange brown, purple, and red), sizes (1 to 100 nm), shapes, and chemical compositions have been reported. In general, AuNPs demonstrate an absorption of peaks between 500 to 550 nm [5]. Several studies depicted that green synthesized AuNPs found a liberal range of potential applications due to their physico-chemical properties along with their catalytic activity, which is different in many ways compared to its bulk form. It can also act as an eco-friendly material because of its reported anti-tuberculosis, anti-fungal, anti-cancer, antibacterial, and antidiabetic activities [6,7].

The plasticizer di(2-ethylhexyl) phthalate (DEHP) used in numerous products starting from food packaging materials, household products, building materials, infusion tubes, toys, baby care, cosmetics, printing inks, pharmaceuticals, blood bags, and pliable-to-rigid plastics, such as polyvinyl chloride, is an endocrine disruptor. [8]. DEHP loosely bound to plastic materials can easily enter into water, air, and soil. It has been proved that DEHP acts as a diabetogenic agent by increasing free radicals and decreasing insulin levels ultimately resulting in loss of pancreatic cells mass [9].

**Figure 1:**
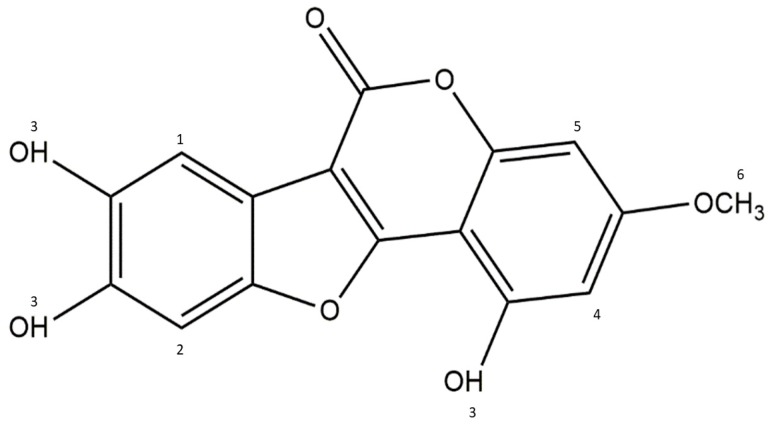
Structure of Wedelolactone.

Phytochemicals are bioactive non-nutrient plant-derived compounds that have been associated with as the treatment of heart disease, arthritis, stroke, diabetes, cancer and have antioxidant properties. Wedelolactone (WDL; 7-methoxy-5, 11,12-trihydroxy-coumestan; C_16_H_10_O_7_; Mol wt: 314.2; Figure 1) is a medicinal plant-derived coumarin isolated from *Wedelia calendulacea* and *Eclipta prostrata* and known for of its therapeutic values in the cure of liver diseases, viral infections, human bronchial epithelial cell injury, and snake bites. It has many pharmacological properties such as anti-inflammatory, anti-cancer, and antioxidant activities [10,11]. Further it inhibits osteoblastogenesis through the NF-κB/c-fos/NFATc1 pathway [12], and reduces lipid levels through AMPK activation [13]. In addition, WDL can suppress the growth of melanoma cells and regulate the cell cycle, via Akt and AMPK pathways [14]. In a recent report, the antidiabetic potential of WDL in a zebrafish model through protecting the pancreatic islets from cytokine-caused apoptosis has been established [15]. However, so far, there was no study conducted and reported with WDL-AuNPs in this regard. Thus, the present study deals with the antidiabetic effect of AuNPs synthesized using WDL on RIN-5F pancreatic cell lines exposed to DEHP toxicity.

## 2. Materials and Methods

### 2.1. Materials and Cell Line

Chloroauric acid was acquired from Aldrich (Mumbai, India) and wedelolactone was obtained from Hong Kong Guokang Bio-Technology Co., Ltd., Baoji, Shan’xi province, China. Ten percent fetal bovine serum (FBS), RPMI-1640, dimethyl sulfoxide (DMSO), phosphate buffer saline (PBS), penicillin, streptomycin, 3-(4,5-dimethylthiazol-2-yl)-2,5-diphenyltetrazolium bromide (MTT), DCFH-DA, acridine orange/ethidium bromide, 1,2-diphenyl-1-picrylhydazyl, nutrient agar, and Mueller Hinton agar were acquired from Himedia, Mumbai, India. The rest of the chemicals and reagents were procured from Fisher Inorganic and Aromatic Limited, Chennai, India S.D Fine Chemical, Mumbai, India.

The RIN-5F cell lines procured from National Center for Cell Science (Pune, India). RIN-5F cell lines were cultured in RPMI 1640 medium enriched with FBS 10%, penicillin (100 U/mL), and streptomycin (100 μg/mL). Cells were grown in an incubator with humidified air containing 95% air and 5% CO_2_ at 37 °C.

### 2.2. Green Synthesis and Characterization of WDL-AuNPs

WDL (1 mg/mL) and auric chloride (1 mM) was taken in different ratios initially. Among the ratios, 2:8 showed a colour change from turbid white to wine red colour, which indicates an appropriate characteristic outcome for the synthesis of AuCl_3_. The WDL-AuNPs were subjected to UV–Vis spectroscopy (Elico SL 196, Hyderabad, India), fluorescence spectrometry (PerkinElmer LS-45, Waltham, MA, USA), X-ray diffraction (XRD) (XRD-6000, Shimadzu, Tokyo, Japan), and dynamic light scattering (Horiba, Kyoto, Japan) analysis. They were further characterized by Fourier transform infrared (FT-IR) spectroscopy, scanning electron microscopy (Hitachi, Japan), transmission electron microscopy (JEOL-JSM 1200EX, Tokyo, Japan), and atomic force microscopy (AFM-Solver Next, NT-MDT, Moscow, Russia). 

### 2.3. Viability Assay

The RIN-5F cells were seeded in tissue culture dishes and employed in the antidiabetic assay in the exponential growth phase. The cells were treated with 625 µM DEHP dissolved in RPMI-1640 and sterile-filtered before use. Cells were grouped into seven experimental groups. Group 1: Normal RIN-5F cells, Group 2: RIN-5F cells exposed with 625 µM DEHP for 24 h, Groups 3, 4, 5, and 6: Treated with 10, 20, 40, and 80 µg/mL of WDL-AuNPs for 24 h, respectively, Group 7: Treated with WDL-AuNPS alone (80 µg/mL for 24 h). Afterward, cells were tested for cytotoxicity, apoptosis, protein expression by western blot, and gene expression by RT-PCR.

Ninety-six-well plates were used to culture RIN-5F at a range (5 × 10^3^ cells/well)/exposed with 625 µM DEHP with different concentrations of WDL-AuNPs. Then, 10 µL of MTT working mixture was added to the RPMI medium, and incubated at 37 °C for 4 h. The formed purple colour formazan was dissolved in 100 µL DMSO and the absorbance was read at 570 nm with a micro-plate reader. 

### 2.4. Glucose Stimulated Insulin Secretion

To assess insulin assay, RIN-5F cells (1 × 10^5^) were added to 6-well plates and incubated with 625 µM DEHP with WDL-AuNPs for 24 h. The cells were exposed to 5 mmol/L glucose for 30 min and stimulated with 25 mmol/L for 30 min. The released insulin content was analyzed using ELISA kit from Millipore, Billerica, MA, USA).

### 2.5. Biochemical Estimations

The PBS suspension of the RIN-5F cell line harvested by trypsinization was used for biochemical studies. The levels of lipid peroxide were determined by the thiobarbituric reactive species assay, which determines the amount of thiobarbituric acid (TBA) reactive malondialdehyde (MDA) [16]. The intensity of the pink colour obtained by the reaction of 2-TBA with lipid peroxidation end products was measured. Lipid peroxidation was also measured by ferrous oxidation–xylenol orange [17]. Superoxide dismutase (SOD) activity was tested by nicotinamide adenine dinucleotide phenazine methosulfate–nitroblue tetrazolium formazan inhibition assay [18]. The catalase activity was studied by dichromate colorimetric assay [19]. The glutathione peroxidase (GPx) activity was studied by the reaction of a certain quantity of enzyme prepared with H_2_O_2_ and GSH for a particular duration [20].

### 2.6. Staining for Apoptosis 

RIN-5F cultured in 6-well plates was incubated with DEHP. The cells were removed after 24 h and washed with PBS buffer and treated with WDL-AuNPs for 24 h. The treated cells were again washed with PBS and stained with DAPI (1 mg/mL) for 30 min at 37 °C. Then they were observed under fluorescence microscopy. Hoechst 33342-stained cells were visualized using a fluorescent microscope fitted with 377–355 nm filter. For apoptosis, acridine orange/ethidium bromide (AO/EB) was added and kept for incubation at 37 °C with 5% CO_2_ for 30 min. The stained cover slip was washed with 1× PBS for removing extra dye. Cells were captured under/by fluorescent microscope (510–590 nm). The intracellular reactive oxygen species (ROS) level was determined [21]. 

### 2.7. Protein Analysis by Western Blot

The cells (RIN-5F) were washed with PBS and detached from culture plates with rapid treatment of trypsin/EDTA. An ice cold RIPA buffer was used to homogenate the samples, then the homogenate was centrifuged for 15 min at 12,000 rpm to remove debris. A total volume of 20 µL (50 µg of protein/well) protein samples were loaded and separated by 10% SDS polyacrylamide gel electrophoresis, then the separated proteins were transferred to PVDF membrane (Millipore, Billerica, MA, USA). The protein containing membranes were blocked with blocking buffer (5% BSA) to minimize non-specific binding sites, then the membranes were incubated with GAPDH (rabbit polyclonal; 1:5000) dilution in 5% BSA in TBST, anti-rabbit Bcl-2, Bax, caspase-3, and caspase-9 (monoclonal; 1:1000), overnight in a gel rocker at 4 °C. Following this, the membranes were incubated with their corresponding secondary antibodies for 1 h at room temperature. The membranes were washed thrice with TBST for 5 min and then the protein bands were visualized by an enhanced chemiluminescence (ECL) method.

### 2.8. RNA Isolation and Reverse Transcription PCR

The total RNA was isolated from WDL-AuNP-treated RIN-5F cells by using RNeasy mini kit reagent (QIAGEN). The isolated RNA was quantified by Nanodrop. A total of 2 µg of RNA was used to analyze RT-PCR. Two-step RT-PCR kit was used to convert cDNA from mRNA template by Oligo(dT), deoxyribonucleotide triphosphate (dNTPs), and reverse transcriptase. All the components were mixed with reverse-transcriptase buffer along with DNA primer for an hour at 37 °C. Once the cDNA conversion was over, the standard PCR was run by gene-specific oligonucleotide primers for IR (224 bp) forward 5′-GCC ATC CCG AAA GCG AAG ATC-3′ reverse 5′-TCT GGG TCC TGA TTG CAT-3′(Pubmed accession number: NM_017071), IRS-1(336 bp) forward 5-GCC AAT CTT CAT CCA GTT GCT-3′ reverse 5′-CAT CGT GAA GAA GGC ATA GGG-3′, GLUT-2 (238 bp) forward 5′-CTC GGG CCT TAG GTG TTC TTC CTT-3′ reverse 5′-TGG TTC CCT TCT GGT CTG TTC CTG-3′ (Pubmed accession number: NM_012879) and β-actin (96 bp) forward 5′-AAG TCC CTC ACC CTC CCA AAA-3′, reverse 5′-AAG CAA TGG TGT CAC CTT CCC-3′ (Pubmed Accession number: V01217;J00691) followed by the initial PCR activation at 95 °C for 5 min. Gel electrophoresis was carried out to measure expression of genes by densitometric scanning. The band intensity of each gene was normalized against β-actin by densitometer (Bio-Rad Lab Inc., Hercules, CA, USA).

### 2.9. Animals 

Male Wistar rats weighing 180 to 220 g were used. The clearance for conducting animal experiments was issued by the Animal Ethics Committee of Adhiparasakthi College of Arts and Science (APCAS), Tamil Nadu, India (Approval number: IAEC/APCAS/02/2017/06). Animals were maintained in polypropylene cages with paddy husk used as bedding, and at a room temperature of 25 ± 2 °C with relative humidity (45 ± 5%) under a 12 h light–dark cycle.

#### 2.9.1. DEHP induction

For successful induction of experimental diabetes, freshly prepared solution of DEHP (100 mg/kg, vehicle-olive oil) was oral administered for 45 days. 

#### 2.9.2. Experimental protocol 

WDL-AuNPs were dissolved in water and different doses of WDL-AuNPs were orally administered with the help of an intragastric tube in the morning for 45 days. A total of 36 rats were categorized into six groups, consisting of a minimum of 6 rats in each group as shown below.

Group I: Control rats 

Group II: WDL-AuNPs (40 mg/kg b.w) alone

Group III: DEHP-induced diabetic control 

Group IV: DEHP + WDL-AuNPs (10 mg/kg b.w) 

Group V: DEHP + WDL-AuNPs (20 mg/kg b.w) 

Group VI: DEHP + WDL-AuNPs (40 mg/kg b.w). 

At the end of the experimental period, the animals were subjected to an overnight fasting and sacrificed by cervical decapitation. Blood samples were collected in tubes containing potassium oxalate and sodium fluoride (3:1) mixture for plasma. The liver tissue was immediately dissected, washed in ice-cold saline to remove the blood, and stored at −80°C for further use. 

#### 2.9.3. Biochemical Assay

Blood glucose level was determined using Accu-Chek Glucometer (Roche diagnostic, Maharashtra, India). The biochemical parameters were measured using Rat ELISA kits based on indirect sandwich enzyme immunoassay, adopting the protocol instructed by the manufacturer (Millipore, St. Charles, MO, USA). Glycogen was assayed by the methods [22] of Morales, Jabbay, and Tenenzi (1975). 

### 2.10. Statistical Analysis

One-way ANOVA was used for statistical analysis at *p* < 0.05.

## 3. Results and Discussion

Natural products from plant origin have the scope to be utilized as natural medicines [23]. Phytocompounds have been a key source for the tremendous growth and development of new drugs, as many of them have entered into clinical trials against various diseases and disorders. Medicinal plants have emerged as the best treatment strategy in the last decades, as an alternative approach to the classical (either physical or chemical) methods. Herein we describe the synthesis of AuNPs using WDL, probably the first ever report. This experiment was designed in view of the integrated approach to find a possible natural protective counteraction against DEHP-induced apoptosis in RIN-5F cell lines.

**Figure 2:**
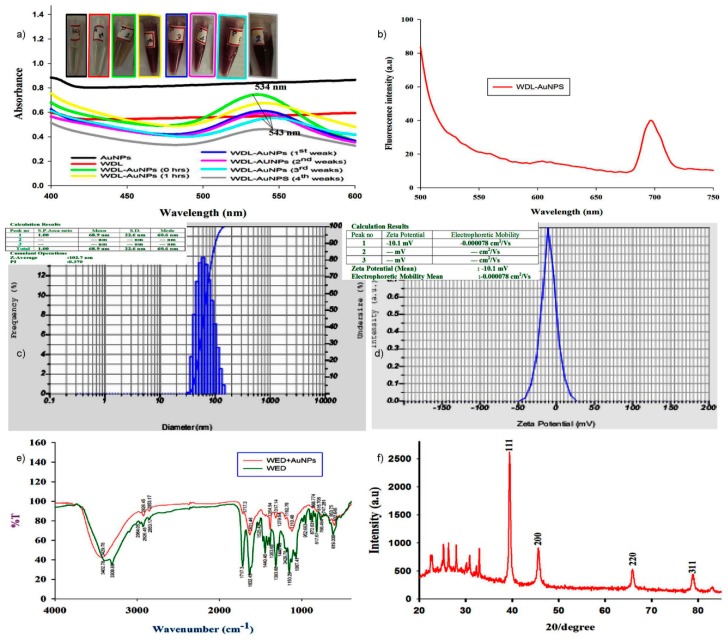
(**a**) Surface plasmon resonance of wedelolactone-gold nanoparticles (WDL-AuNPs) synthesized by WDL upon reduction of AuCl_4_ observed by UV–Vis spectroscopy. (**b**) Emission spectra of AuNPs. (**c,d**) DLS and Zeta potential analysis of synthesized WDL-AuNPs. Mean size distribution of WDL-AuNPs was found to be 102.7 nm. WDL-AuNPs slightly increased the negative surface charge from –10.1 mV. (**e**) FT-IR absorption spectra of pure WDL and WDL-functionalized AuNPs. (**f**) XRD patterns of the WDL-functionalized AuNPs. The XRD pattern was obtained according to four planar angles ranging from 20 to 80 at 20 peak values of 38.43°, 45.66°, 65.80°, and 78.76°, respectively, of 111, 200, 220, and 311 planes for gold.

In the UV-Vis spectra (Figure 2a) of the WDL-AuNPs samples, a strong absorbance was noted between 534 to 540 nm. This band was not present in the spectrum of WDL, confirming the reduction of gold ions to metallic gold and the formation of gold nanoparticles. All the AuNP samples were stable up to a period of one month. The change in peak intensities can be correlated with color change—from mild yellow to red wine. Further studies were carried out with an 8:2 ratio of synthesized AuNPs. Our results conform to the reports of Hwang et al. [24], showing the chlorogenic acid oxidation in NPs which has favored the gold ions reduction. The samples were also incubated for different time intervals to analyze their stability. Figure 2b presents the data of photoluminescence of WDL-AuNPs. The emission spectra were characterized by a sharp peak at 680 nm that attributed to quantum confinement of electrons from AuNPs. 

The colloidal stability of NPs was examined using zeta potential analysis. The average particle size was found to be about 102.7 nm with a good zeta potential of −10.1 mV for the WDL-AuNPs (Figure 2c,d). This confirms the stability of the colloids [25]. This stipulates that the obtained AuNPs had a good a repulsive electrostatic force, which provides the mono dispersity of the particles. 

The FT-IR spectra used to identify the various functional groups that take part in the bioreduction and stabilization of AuNPs are shown in Figure 2e. The individual participants in the formation of nanostructures with WDL are depicted. The peak appearing in the spectrum of WDL at 3402.78 cm^−1^ is due to the of O−H stretch of carboxylic acids. The peaks at 2926.45 and 1717.3 cm^−1^ are attributed to C−H and C–O (ketone) stretching vibrations that belong to ketones. The peak at 1314.25 cm^−1^ can be attributed to C–C stretching vibration. In the IR spectrum of the WDL-AuNPs, the peak due to O–H stretching shifted to 3429.78 cm^−1^. Similarly, the other peaks observed at 2926.45, 1192.76, 1525.42, and 1440.46 cm^−1^ in the IR spectrum of WDL completely disappeared in the WDL-AuNPs samples. Short peaks that appeared at 617.67 to 872.631 cm^−1^ in the spectrum of WDL experienced a small shift in the IR spectra of WDL-AuNPs. All these results show that these groups were involved in the capping and stabilization of AuNPs. The crystallinity of the biosynthesized AuNPs was inspected by XRD analysis [26]. The typical XRD pattern of WDL-AuNPs is exhibited in Figure 2f, illustrating the crystalline nature of synthesized NPs. In the XRD pattern, five diffraction peaks at 2θ values of 38.43°, 45.66°, 65.80°, and 78.76°, corresponding to (111), (200), (220), and (311) crystal planes of the face-centered cubic crystal system, respectively, are seen. These results are in parallel with reported values for similar Au nanostructures [27]. EDX spectrum strongly supports the presence of gold ions. The peaks produced from the analysis match well with the elemental composition of the sample. Similar diffraction patterns have been previously reported for AuNPs synthesized using curcumin as a reducing and capping agent [28]. Thus, it is evident that WDL acts as a strong reducing agent by altering free radicals to form WDL-AuNPS. Thus, NPs produced using WDL can be used as an alternative for commercial products to treat the harmful responses arising from reactive oxygen species (ROS) [29].

**Figure 3:**
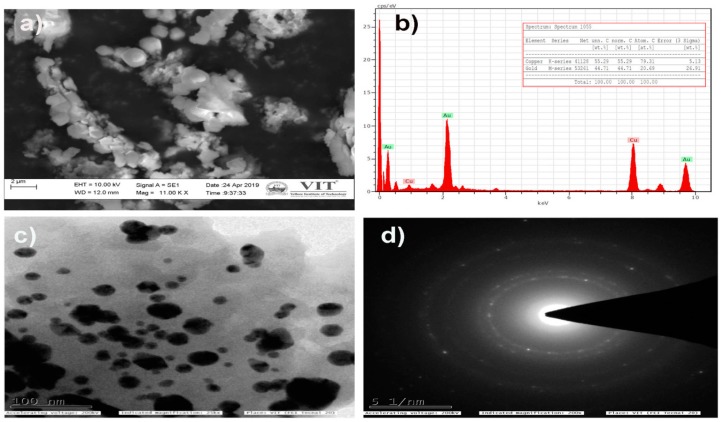
(**a**) Scanning electron microscope (SEM) and (**b**) Energy-dispersive X-ray (EDX) profile of WDL-AuNPs. (**c**) Transmission electron microscope (TEM) micrograph of the 1 mM AuCl^4−^ ions with WDL showing synthesized AuNPs. Purified NPs from WDL were examined by electron microscopy. The range of observed diameters of the synthesized AuNPs was about 20 to 50 nm. (**d**) Selected area diffraction (SAED) pattern.

The SEM and TEM analysis were performed to study the surface morphology and size of the WDL-AuNPs, along with the EDX analysis. From the histograms illustrated in Figure 3c,d, it can be noted that WDL-AuNPs were at a size of 50 nm during the synthesis, with different particle morphology, such as spherical, oval, and triangular. Biomolecules aid the formation of different shaped NPs because of the uncontrolled reduction sites present in them. The AuNPs exhibited an average size of 40 nm according to SEM (Figure 3a). The presence of metallic Au ions in the synthesized sample was further confirmed by the EDX analysis. The EDX spectral (Figure 3b) studies showed strong peaks of metallic gold in the range of 3 to 4 KeV.

**Figure 4:**
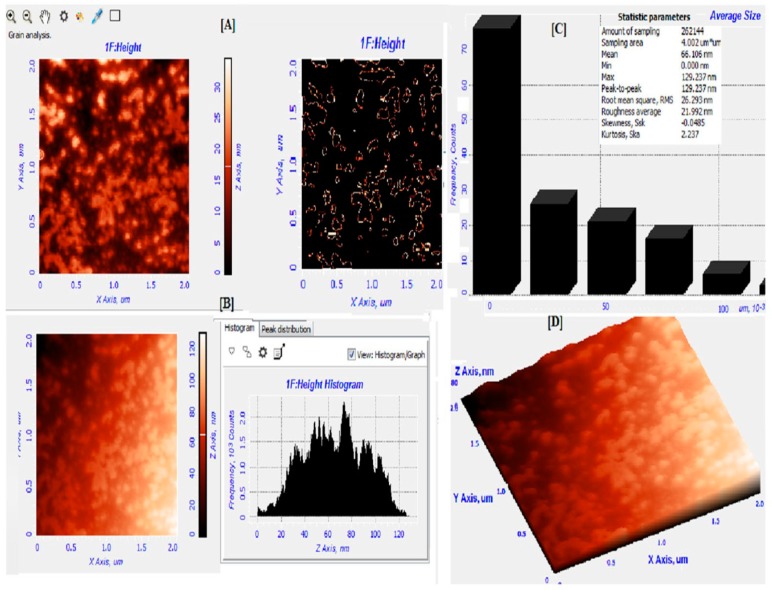
Atomic force microscopy (AFM) analysis of gold nanoparticles. (**A**) Grain analysis of AuNPs in 2D image. (**B**) Histogram of peak distribution of AuNPs. (**C**) Average size analysis. (**D**) 3D image of AuNPs.

To find out the surface morphology and topology of biosynthesized AuNPs, we analyzed them using an atomic force microscope. The results indicate that the AuNPs were spherical and some particles varied in shape and size. The size distribution of the AuNPs varied from 30 to 105 ± 5 nm in size, and the average grain size was detected to be 66.10 ± 5 nm (Figure 4A–D). Further, we also carried out analysis with 3D image of AuNPs to find out the distribution of different sizes of biosynthesized AuNPs. The results clearly indicate that the size of AuNPs range from 65 to 77 ± 5 nm (Figure 4C,D). During the synthesis, bigger particles were also observed due to agglomeration.

**Figure 5:**
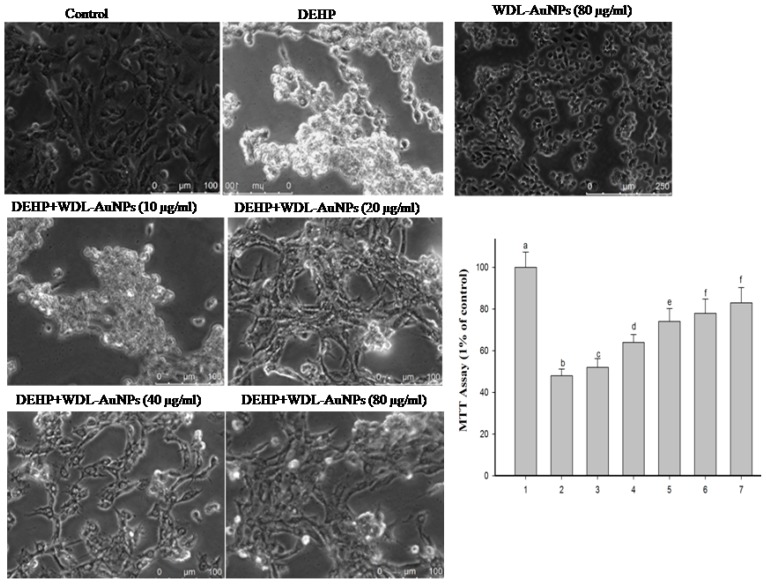
Effect of WDL-AuNPs on cell viability in DEHP-induced RIN-5F pancreatic β-cells. (1) Normal RIN-5F cells, (2) DEHP (625 µM), (3) DEHP + WDL-AuNPs (10 µg/mL), (4) DEHP + WDL-AuNPs (20 µg/mL), (5) DEHP + WDL-AuNPs (40 µg/mL), (6) DEHP + WDL-AuNPs (80 µg/mL), (7) WDL-AuNPs (80 µg/mL). Values were mentioned as mean ± SD for all the three experiments in every group. The values which do not share a common letter (a–f) are predominantly significant in difference over the groups (*p* < 0.05).

**Figure 6:**
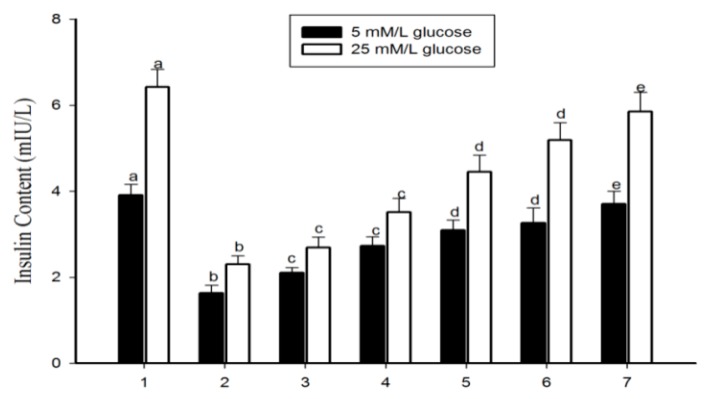
Effect of WDL-AuNPs on insulin secretion in RIN-5F cells. (1) Normal RIN-5F cells, (2) di(2-ethylhexyl) phthalate (DEHP) (625 µM), (3) DEHP + WDL-AuNPs (10 µg/mL), (4) DEHP + WDL-AuNPs (20 µg/mL), (5) DEHP + WDL-AuNPs (40 µg/mL), (6) DEHP + WDL-AuNPs (80 µg/mL), (7) WDL-AuNPs (80 µg/mL). Value is mentioned as mean ± S.D. for all the three experiments in every group. ^a–e^ denotes that the values which do not share a common letter are predominantly significant in difference over the groups (*p* < 0.05).

Diabetes mellitus is represented by a comparatively low or complete deficiency of insulin secretion by β-cells. In due course, over-secretion of insulin may arise leading to pancreatic β-cell failure [30]. Pancreatic β-cells play a crucial role in the alimentation of the glucose equilibrium; therefore, we used RIN-5F cells (a rat islet cell line) to examine the levels of insulin. The direct exposure of DEHP to insulin-secreting pancreatic β-cells causes intracellular ROS-mediated apoptosis, and induces the ER stress ultimately affecting insulin secretion from pancreatic β-cells [9]. The cytotoxicity assay revealed that exposure to DEHP (625 mmol/L) for 24 h significantly reduced RIN-5F cell viability, compared to the normal cell line group. The treatment with WDL-AuNPs (10, 20, 40, and 80 µm/L) for 24 h showed an improvement in cell viability (Figure 5). The present study also explores the cytotoxic effects of DEHP on the insulinoma RIN-5F cells and the molecular mechanisms behind the process. WDL-AuNPs treatment to DEHP-exposed cells induced the secretion of insulin in a dependent way and restricted the deleterious responses caused by DEHP. The significant effects were observed at 10, 20, 40, and 80 µm both in low and high glucose media environment (Figure 6).

Oxidative stress can be regarded as one of the causative agents for diabetes mellitus, insulin resistance, and cardiovascular disorders [31]. DEHP has the potential to decompose the intracellular antioxidants and alter the sequential events involved in the lipid peroxidation process, which show strong relation with oxidative stress. People with high insulin resistance indices along with altered metabolism are highly sensitive to DEHP exposure [32]. Evidences from various studies confirmed that DEHP cause pancreatic dysfunction through the induction of oxidative stress (MDA); alterations in the enzymatic and non-enzymatic antioxidant defense (SOD, catalase, and glutathione peroxidase) accompanied by elevated ROS production and DNA damages in the pancreatic tissue [33,34,35]. Our results showed high levels of lipid peroxidation and significant decrease in enzymatic antioxidant levels in the DEHP-exposed RIN-5F cell line. DEHP-induced WDL-AuNP-treated cells progressively decreased the levels of TBARS and restored enzymatic antioxidant enzymes (Table 1).

**Figure 7:**
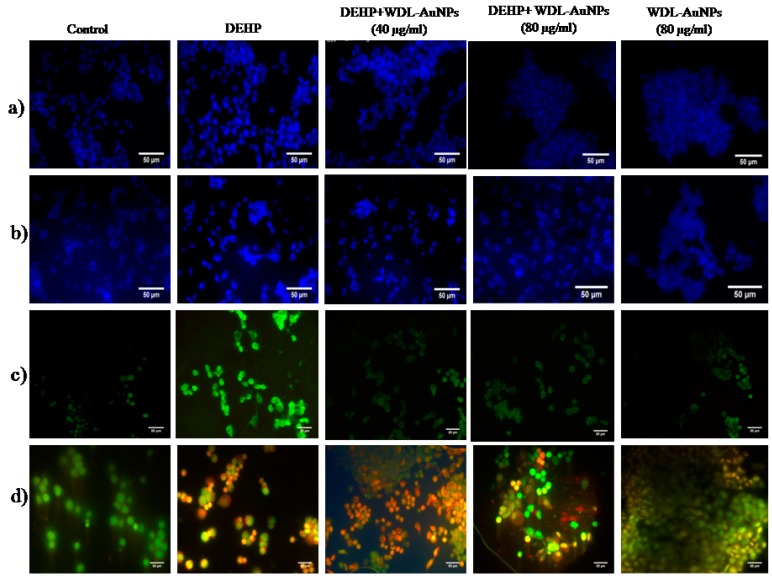
Effect of WDL-AuNPs on DEHP-induced apoptotic features in RIN-5F cells. (**a**) DAPI stain, (**b**) Hoechst 33342, (**c**) ROS, (**d**) AO/EB stain.

Apoptotic cells showcase the condensation of the nucleus that can be experimentally studied by DAPI staining; a fluorescent staining that distinguishes the nuclear damage or chromatin condensation [36]. Being an environmental toxicant, DEHP exposure has increased the apoptotic factors, such as nucleolus and chromatin condensation, in RIN-5F cells. Figure 7a shows the reduced nuclear fragmentation and percentage of apoptotic cells in WDL RIN-5F cells. In addition, treatment with WDL-AuNPs for 24 h reduces the apoptotic features and modulates the nuclear fragmentation, and thereby reduces the percentage of apoptotic cells.

WDL-AuNPs treatment showed significant reduction of nuclear fragmentation in DEHP-induced apoptotic RIN-5F cells (Figure 7b). Intracellular ROS levels were estimated by DCFH, a ROS sensitive fluorometric test, to certify the roles of ROS generation in the DEHP-induced RIN-5F cell apoptosis. The DCFH is a non-polar and non-fluorescent compound that can enter cells. Sun et al. confirmed the detrimental effect of DEHP and MEHP in altering the mitochondrial membrane potential and the releasing of lysosomal enzymes, thereby enhancing the formation of ROS [9]. The observations reveal that DEHP exposure could elevate ROS production, which increases the green fluorescence under the microscope (Figure 7c), whereas the DEHP-exposed RIN-5F cells treated with WDL-AuNPs showed significant decrease in ROS levels. 

The staining results were studied using fluorescence microscopy, and AO/EB staining differentiates between live, apoptotic, and necrotic features of the cells. Figure 7d reveals that the normal RIN-5F cell line contained normal nuclear chromatin and appeared green, whereas DEHP-exposed cells showed condensed nuclei and apoptotic bodies’ exhibited orange-to-red nuclear staining. DEHP with WDL-AuNP-treated cells showed reduced apoptotic changes (Figure 7d).

**Figure 8:**
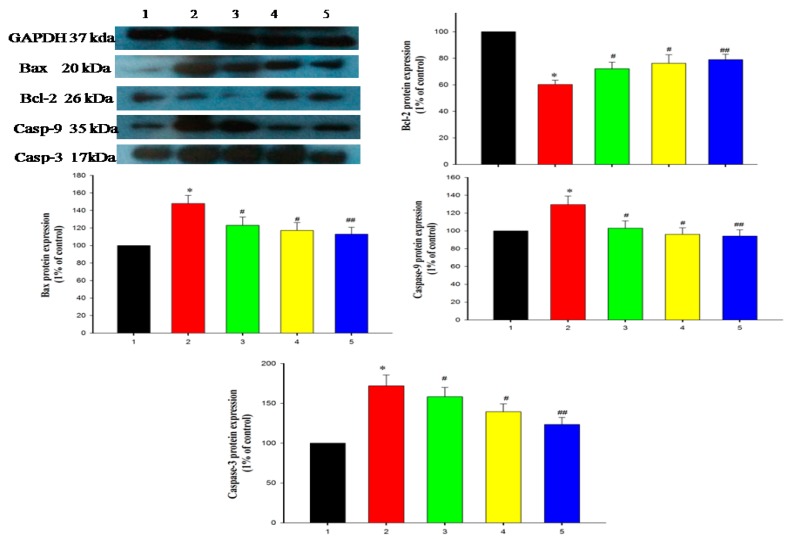
Effect of WDL-AuNPs on the protein expression of Bcl-2 and Bax in RIN-5F cell lines. The graph indicates the quantification results normalized to GAPDH levels. (1) Normal RIN-5F cells, (2) DEHP (625 µM), (3) DEHP + WDL-AuNPs (40 µg/mL), (4) DEHP + WDL-AuNPs (80 µg/mL), (5) WDL-AuNPs (80 µg/mL). Values are mentioned as means ± SD of three independent tests. * Compared with control cells group (*p* < 0.05); ^#, ##^ Compared with the DEHP cells group (*p* < 0.05).

The mitochondrial-dependent apoptosis pathway is triggered by the production of cyto-c in the mitochondria (maintains equilibrium between anti-apoptotic Bcl-2 family proteins and pro-apoptotic proteins). Thus, it promotes cell survival by interrupting Bcl-2: Bax ratio (Bax induces cell apoptosis) [37]. The well-known pro-apoptotic factors include Bax and Bad controlled by Bcl2 and cl2110, the DEHP-induced apoptotic pathway, BCL_2_, led to cause a defect in the pancreatic cell [38]. Bax-associated protein molecules drive the permeabilization of the outer mitochondrial membrane, which leads to the production of cyto-C, followed by binding of dATP and APAF-1, to form a multimeric complex that recruits pro-casp-9, activates the caspases cascade proteins, and executes cell apoptosis [39]. In our study, the apoptotic signaling cascades, such as cyto-c, Casp-3, -9, and Bax, were significantly increased in DEHP-exposed pancreatic β-cell lines. Consequently, Bcl-2 expression was suppressed in the DEHP-exposed RIN-5F cell line when compared to control cell lines (Figure 8). WDL-AuNP-treated RIN-5F modulates DEHP-induced apoptotic cell signaling cascades. Peng and Zhang recently showed that WDL elevates the proapoptotic factors Bad and Bax and APAF-1 triggered the casp-3 and casp-9 to get expressed, which further inhibits the anti-apoptotic agents Bcl2 and Bcl-XL [14].

**Figure 9:**
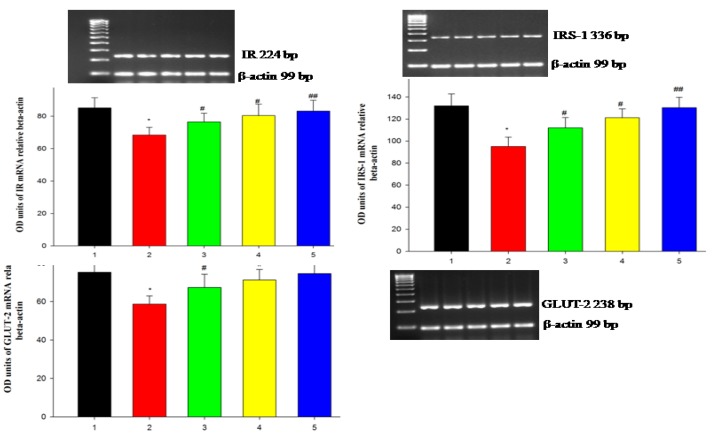
WDL-AuNPs prevented the activation insulin-signaling proteins on DEHP-induced RIN-5F cells in mRNA expression. (1) Normal RIN-5F cells, (2) DEHP (625 µM), (3) DEHP + WDL-AuNPs (40 µg/mL), (4) DEHP + WDL-AuNPs (80 µg/mL), and (5) WDL-AuNPs (80 µg/mL). The expressions of insulin receptor (IR), insulin receptor substrate-1 (IRS-1), and glucose transporter-2 (GLUT2) were analyzed by RT-PCR. Values are mentioned as means ± SD of three independent tests. * Compared with control cells group (*p* < 0.05); ^#, ##^ Compared with the DEHP cells group (*p* < 0.05).

DEHP-induced pancreatic cells cause dysfunction in pancreatic β-cells and also impede the metabolism of carbohydrates, thus decreasing levels of glucose uptake [40]. DEHP-mediated deduction in the insulin receptor is conceded with the reduced IRS-1. It has been proven that ROS could degrade IRS-1 [41]. Acetylation of IRS is tolerant of tyrosine phosphorylation and simplifies the insulin-stimulated signal transduction proteins by activating P13K, phosphorylation, and activation of AKT. This results in the translocation of AKT to the intracellular membranes, thereby preventing apoptosis in neuronal cells, and imparts cell survival [41]. The plasma membrane-bound GLUT2 protein level decreased drastically in the DEHP-treatment. In DEHP-induced condition, there was decreased insulin. As a result, significant decrease in insulin-signaling molecules, such as IR, IRS-1, Akt, and GLUT2 in the pancreas [42]. In our study, the insulin-signaling pathway proteins, such as IR, IRS-1 and GLUT2, were relatively low in the DEHP-induced RIN-5F cell line, indicating the establishment of apoptosis as a result of DEHP. Further, treatment with the WDL-AuNPs cell line showed up-regulated expression of IR, IRS-1, and GLUT2 in the RIN-5F cell line (Figure 9). Thus, it could be suggested that WDL-AuNPs recovered insulin sensitivity by activating insulin signal molecules in pancreatic cells, and promoting high glucose uptake to treat diabetes.

Insulin mediates the entry of glucose from the blood into tissues as an immediate source of energy, with excess glucose stored as glycogen or fat in target tissues, such as liver, adipose tissue, and skeletal muscle [43]. The circulating ratio of insulin was significantly diminished in the DEHP-induced diabetic rats. Most of the reports represent that DEHP administration decreases the serum insulin levels in rat [44]. Treatment of WDL-AuNPs moderately improved the insulin level. The 40 mg dose of WDL-AuNPs showed a near normal level of insulin, which might be through disturbing the DEHP-induced adverse effect on the beta cells of pancreas. 

Increased amount of fasting blood glucose was observed in the DEHP-induced diabetic rats. It was obvious that the DEHP exposure impaired β-cell function and caused insulin resistance-induced hyperglycemia in rats [41,45]. As a result, there was elevated fasting blood glucose levels in DEHP treated rats. WDL-AuNPs administration dose-dependently reduced the fasting blood glucose level by nullifying the adverse effect of DEHP on pancreas and improving the insulin sensitivity in the target tissues through its anti-oxidant and free radicals scavenging activities [46]. Administration of WDL-AuNPs (40 mg/kg bw) was effective against the DEHP-induced hyperglycemia when compared to other doses WDL-AuNPs (10, 20 mg/kg bw) (Table 2). It might be due to increased insulin sensitivity in target tissues. DEHP exposure decreased the glycogen content in the liver. Previous studies also stated that DEHP impairs insulin-signaling and reduces the glycogen level in liver and adipose tissue by imparting insulin resistance and excess glycogenolysis [47,48]. On the contrary, WDL-AuNPs treatment showed the dose-dependent increase in glycogen level in liver of DEHP administered diabetic rats through improving the insulin level and, thereby, the glycogen storage in the liver [46]. 

## 4. Conclusions

In conclusion, AuNPs were synthesized by bottom-up processes through a suitable, eco-friendly, simple, and non-toxic approach by using WDL as a reducing and stabilizing agent. Characterization studies showed that the WDL-AuNPs have morphological parameters suitable for medicinal applications. The WDL-AuNPs protect RIN-5F cells from DEHP-exposed toxicity by increasing cell viability and insulin secretion. The AuNPs also prevent the cells from oxidative damage and normalize the regulation of Bcl-2 family proteins through an unregulated insulin-signaling pathway. Taken together, this finding suggested that WDL-AuNPs may potentially enhance the therapeutic target for β-cell survival and anti-diabetic activity.

## Figures and Tables

**Table 1:** Effect of WDL-AuNPs on thiobarbituric acid reactive substances (TBARS) and antioxidant levels in the DEHP treated RIN-5F cell lines.

Enzymes	Normal Cells	DEHP (625 µM)	DEHP + WDL-AuNPS (40 µg/mL)	DEHP + WDL-AuNPS (80 µg/mL)	WDL-AuNPS (80 µg/mL)
**TBARS** **(nmol/mg protein)**	1.50 ± 0.10 ^a^	5.52 ± 0.44 ^b^	3.36 ± 0.26 ^c^	2.10 ± 0.17 ^d^	1.72 ± 0.15 ^ad^
**SOD** **(U** **∗/mg of protein)**	65.01 ± 4.05 ^a^	23.8 ± 1.9 ^b^	45.89 ± 4.45 ^c^	59.10 ± 5.13 ^a^	62.01 ± 3.85 ^a^
**CAT** **(U** **∗∗/mg of protein)**	1.5 ± 0.10 ^a^	0.18 ± 0.01 ^b^	0.72 ± 0.06 ^c^	0.98 ± 0.08 ^d^	1.4 ± 0.12 ^a^
**GPx** **(U^#^ /mg of protein)**	4.2 ± 0.35 ^a^	1.87 ± 0.15 ^b^	3.12 ± 0.25 ^c^	3.5 ± 0.28 ^c^	3.8 ± 0.29 ^a^

Value is given as mean ± S.D. for triplicates in each group. Values not sharing a common superscript differ significantly (a–d) at *p* < 0.05 (DMRT). U * = the concentration of enzyme needed for inhibiting the chromogen released by 50% in 1 m under standard condition. U ** = µmole of hydrogen peroxide degraded/min. U**^#^** = µmole of GSH used/min. SOD: Super oxide dismutase activity; CAT: Catalase activity; GSH-px: Glutathione peroxidase activity.

**Table 2:** Effect of WDL-AuNPs on glucose, insulin, and liver glycogen in normal and diabetic rats.

Group	Glucose (mg/dL)	Insulin (µU/mL)	Glycogen (mg/100g Tissues)
Control	102.01 ± 9.25 ^ad^	11.61 ± 1.09 ^a^	51.94 ± 4.30 ^a^
WDL-AuNPs (40 mg/kg bw)	94.81 ± 8.41 ^a^	10.98 ± 0.91 ^a^	53.01 ± 3.51 ^a^
DEHP	190.44 ± 15.65 ^b^	5.16 ± 0.85 ^b^	24.34 ± 2.01 ^b^
DEHP + WDL-AuNPs (10 mg/kg bw)	170.63 ± 13.54 ^c^	6.44 ± 0.45 ^c^	35.11 ± 2.89 ^c^
DEHP + WDL-AuNPs (20 mg/kg bw)	165.84 ± 12.54 ^c^	7.09 ± 0.78 ^cd^	39.18 ± 3.14 ^d^
DEHP + WDL-AuNPs (40 mg/kg bw)	120.91 ± 11.69 ^d^	8.11 ± 0.69 ^d^	43.79 ± 4.29 ^d^

Values are given as means ± S.D. for six rats in each group. Values not sharing a common superscript differ significantly (a–d) at *p* ≤ 0.05 Duncan’s Multiple Range Test (DMRT).
